# Aftercare Provision for Bereaved Relatives Following Euthanasia or Physician-Assisted Suicide: A Cross-Sectional Questionnaire Study Among Physicians

**DOI:** 10.3389/ijph.2024.1607346

**Published:** 2024-07-25

**Authors:** Sophie C. Renckens, H. Roeline Pasman, Agnes van der Heide, Bregje D. Onwuteaka-Philipsen

**Affiliations:** ^1^ Department of Public and Occupational Health, Amsterdam UMC, Amsterdam, Netherlands; ^2^ Expertise Center for Palliative Care Amsterdam UMC, Amsterdam, Netherlands; ^3^ Department of Public Health, Erasmus MC, University Medical Center, Rotterdam, Netherlands

**Keywords:** medical aid in dying, support needs, family, bereavement care, cross-sectional questionnaire

## Abstract

**Objectives:** Relatives of patients who died after euthanasia or physician-assisted suicide (EAS) might need (specific) aftercare. We examined if and how physicians provide aftercare to bereaved relatives of patients who died after EAS, and which patient-, physician- and process characteristics are associated with providing aftercare.

**Methods:** A cross-sectional questionnaire study was conducted among 127 physicians (general practitioners, clinical specialists, and elderly care physicians) in the Netherlands. Associations were examined using multivariable logistic regression analyses.

**Results:** Most physicians had had at least one follow-up conversation with bereaved relatives (77.2%). Clinical specialists less often provided aftercare compared to GPs. Also, aftercare was more often provided when the deceased had a cohabiting partner. Topics addressed during aftercare conversations included looking back on practical aspects of the EAS trajectory, the emotional experience of relatives during the EAS trajectory and relatives’ current mental wellbeing. A minority of aftercare conversations led to referral to additional care (6.3%).

**Conclusion:** Aftercare conversations with a physician covering a wide-range of topics are likely to be valuable for all bereaved relatives, and not just for “at risk” populations typically targeted by policies and guidelines.

## Introduction

Death of a loved one is a significant life event, also if a loved one died after euthanasia or physician-assisted suicide (EAS). Euthanasia is the intentional termination of a patient’s life at their explicit request by a physician who administers the lethal medication, while in physician-assisted suicide the patient self-administers the lethal medication prescribed by a physician. In the Netherlands, EAS is allowed since 2002 if physicians adhere to the legal due care criteria, i.e., they must 1) be satisfied that the patient’s request is voluntary and well-considered, 2) be satisfied that the patient’s suffering is unbearable and without prospect of improvement, 3) have informed the patient about his situation and prognosis, 4) have come to the conclusion, together with the patient, that there is no reasonable alternative, 5) consult at least one other, independent physician (e.g., a specially trained SCEN physician), and 6) exercise EAS with due medical care and attention. Adherence to the due care criteria is assessed by a regional Euthanasia Review Committee after the EAS is performed [1]. In 2021, approximately 9,000 people died after EAS in the Netherlands, accounting for 5.4% of the total deaths [2]. Most of these people leave behind one or more relatives that might need aftercare to cope with the death of their loved one.

The Dutch Royal Medical Association (KNMG) describes in their most recent position paper on EAS that aftercare for bereaved relatives following EAS is important, and not different for EAS than for natural death, but refrain from explaining what such aftercare should entail [3]. Moreover, (inter)national and local guidelines on general bereavement care are scarce, but several researchers have tried to formulate bereavement care principles and standards. Such standards, for example, suggest that bereavement care should be provided according to need and with extra care for those “at risk.” It should include information about grief experiences and available bereavement services [4, 5]. While many guidelines and studies focus on grief, previous research in the ICU and in palliative care units showed that many relatives wish to discuss more practical aspects of the end-of-life trajectory, rather than mental wellbeing [6, 7]. We have therefore decided to focus on the broader concept of aftercare, which also addresses more general support. Bereavement care may then be seen as an aspect of aftercare, that centers on (complicated) grief.

Regarding EAS, previous research shows that bereaved relatives of people who died after EAS report similar or lower levels of grief compared to relatives who experienced a natural loss [8–11]. However, it is likely that relatives may have specific questions about the performed EAS, as the KNMG also acknowledges [3]. It is not uncommon that complexities arise during the EAS trajectory such as, lack of relatives’ support for the patient’s EAS request, sudden acceleration of the EAS trajectory due to rapid deterioration of the patient’s health, and moving a patient to another place to perform EAS [12]. Therefore, bereaved relatives may need an aftercare conversation with the physician about the performed EAS to discuss these complexities and assess the need for further aftercare. According to the Dutch law, physicians are responsible for the entire EAS trajectory (e.g., nurses have no formal role) and they may thus be the most suitable healthcare professionals to provide this aftercare.

A mortality follow-back survey of patients who died non-suddenly in several European countries showed that the number of cases in which general practitioners (GPs) contact relatives of patients with regard to bereavement counselling, varies between 64% (Belgium) and 93% (Netherlands) [13]. Little research has been conducted on physicians providing aftercare for bereaved relatives following EAS. A recent interview study by Boven et al. described that most physicians in Belgium give their contact details to relatives right before or after EAS, so that relatives can contact them if needed [14]. They rarely initiate post-loss contact themselves. If relatives do not contact them they consider this as a confirmation that the EAS trajectory went well [14]. However, people that are at risk for developing grief-related disorder are less likely to seek support themselves [15]. Also, a review of studies in the palliative care setting demonstrated that while bereavement care is integral to palliative care according to the WHO definition, it is under-researched and typically insufficiently resourced nor systematically implemented [4].

To the best of our knowledge, it is unknown to what extent and in which cases aftercare is provided by physicians for bereaved relatives following EAS and what such aftercare entails exactly. Therefore, we examined how many physicians provide aftercare to bereaved relatives of patients who died after EAS, what this aftercare entails and which patient-, physician- and process characteristics are associated with providing aftercare.

## Methods

### Design and Population

As part of the fourth evaluation of the Dutch euthanasia act [2], we conducted a retrospective cross-sectional questionnaire study among physicians in the Netherlands. In total, 1,100 GPs, 1,000 clinical specialists (cardiologists, pulmonologists, internists, neurologists, surgeons and intensive care physicians, all working in the hospital) and 400 elderly care physicians were invited to participate. In the Netherlands, elderly care physicians primarily work in nursing homes and are the first point of contact for medical concerns in this setting. GPs, the selected clinical specialists and elderly care physicians are involved in the great majority of deaths in the Netherlands (estimation: 95%). The sample was drawn taking into account the extent to which physicians in different specialties deal with deaths and medical end-of-life decisions. Eligible participants were physicians who had worked in adult patient care in the Netherlands in the past year.

### Data Collection

Data were collected between April and September 2022. Eligible physicians received a written invitation with information about the questionnaire study, a personal log-in code and a link to the online questionnaire. If the physicians logged-in they were asked to consent to participate. If they consented they were given access to a separate website with the questionnaire. This ensured anonymity without precluding the possibility of sending two reminders to non-responders. The last reminder included an abbreviated 2-page questionnaire on paper, which did not include the questions that are of interest for the current study. Addresses were obtained from a national database of registered physicians (IQVIA).

### Measurements

The questionnaire used in this study was largely the same as the questionnaire used in the previous three evaluations of the euthanasia act [16–18] (Supplementary Material S1). The questionnaire included questions about the following subjects: a) physician’s characteristics (demographic and professional characteristics), b) experience with EAS requests and their performance, c) characteristics of the most recent explicit EAS request in the past 5 years and d) physician’s opinion on several statements. Part c included different versions, namely, about an EAS request of a person with 1) dementia; 2) an accumulation of health problems related to old age; and 3) another condition. Based on their experience with these particular conditions, physicians were directed to one of the cases and only completed the case to which they were directed. Physicians were only directed to the case of a patient with another condition if they did not have experience with a patient with dementia or an accumulation of health problems related to old age in the past 5 years (Supplementary Material S2).

In this study, the focus is on part c of the questionnaire, particularly on aftercare for bereaved relatives. The questions about aftercare were not included in previous evaluations of the euthanasia act. Three questions related to physicians’ experiences with aftercare for bereaved relatives of patients who died after EAS were included: 1) did you have an aftercare conversation about the euthanasia or physician-assisted suicide with the relatives (yes, once/yes, multiple times/no); 2) If yes, what was discussed in this aftercare conversation(s) (open-ended question); 3) If yes, did this lead to referral to additional care? (yes, namely; no).

This study also made use of the questions regarding the patient, physician and process characteristics, including patient’s age (in years), main diagnosis (cancer/dementia/accumulation of health problems related to old age/other), level of dependency (independent/limited care dependent/care dependent), life expectancy (<1 month, 1–5 months, 6–12 months, >12 months), physician’s medical specialty (GP/clinical specialist/elderly care physician), physician’s gender (male/female/other), physician’s age (in years), physician’s religion (yes/no), physician’s work experience (in years), opinion of relatives about EAS request (neutral/supportive/not supportive), duration of EAS decision-making process (<1 month/1–3 months/>3 months), performed euthanasia or physician-assisted suicide (euthanasia/physician-assisted suicide), complications in EAS (yes/no).

### Data Analysis

Statistical analyses were performed with SPSS IBM 28. Descriptive statistics were used to summarize background characteristics of the physicians. The two categorical items on aftercare for bereaved relatives were also summarized using descriptive statistics. In addition, the open-ended question on topics discussed in the aftercare conversations were categorized and analyzed with descriptive statistics. In the results we show examples of answers physicians wrote down in the text entry field. Finally, univariable and multivariable logistic regression analyses were performed to explore which variables are associated with having had an aftercare conversation with bereaved relatives. Independent variables tested for associations were patient’s age, main diagnosis, level of dependency, life expectancy, physician’s specialty, physician’s gender, physician’s age, physician’s religion, physician’s work experience, opinion of relatives about EAS request, duration of EAS decision-making process, performed euthanasia or physician-assisted suicide, complications in EAS. All variables with a *p*-value of less than 0.10 in univariable logistic regression analyses were included in the multivariable logistic regression analysis, where a *p*-value <0.05 was considered statistically significant.

## Results

Of the 2,500 physicians invited, 245 did not meet the inclusion criteria [e.g., not working at the address (anymore) (n = 137), not worked in adult patient care in past year (n = 83), other (n = 25)]. Of the 2,255 eligible physicians, 746 responded (33%). Of the non-responders (n = 1,509), 39 provided a reason for non-response, with lack of time (n = 28) being the most reported reason. Of the 746 physicians who completed the questionnaire, 476 completed the extensive online questionnaire. A total of 258 physicians had received an explicit request for EAS in the past 5 years and filled in questions about their most recent case, of whom 131 granted the request and performed euthanasia. Only data from physicians who indicated that there were relatives involved in the EAS trajectory and had data on aftercare were included in the analyses (n = 127).

The physicians’ background characteristics are shown in Table 1. Of the physicians (n = 127), 71.4% were general practitioner, 10.3% clinical specialist, and 18.3% elderly care physician. Furthermore 51.2% were male, 27.6% were religious, 8.7% were palliative care consultants and/or members of a palliative care team and 7.9% were SCEN physicians. Furthermore, physicians had a median age of 52 years and a median amount of work experience of 22 years.

**TABLE 1:** Background characteristics of physicians from the questionnaire (n = 127) (Fourth evaluation of the Dutch euthanasia act, Netherlands, 2022).

	Total N (%)
Demographics	
Gender	
Male	65 (51.2%)
Female	61 (48.0%)
Other	1 (0.8%)
Age (years)	
Median (IQR)	52.0 (15.0)
Religious belief^a^	
Yes	35 (27.6%)
Professionals Characteristics	
Medical specialty	
General practitioner	90 (71.4%)
Clinical specialist	13 (10.3%)
Elderly care physician	23 (18.3%)
Years of working experience	
Median (IQR)	22.0 (16.0)
Consultant palliative care/member palliative care team	
Yes	11 (8.7%)
SCEN physician^b^	
Yes	10 (7.9%)

Missing values: age 1, medical specialty 1, years of working experience 1, consultant palliative care/member of palliative care team 1, SCEN physician 1.

^a^ According to the respondent, Christian religion in 96.9% of the cases.

^b^ A SCEN physician is a trained physician from whom other physicians can obtain information and advice about EAS, or request a formal consultation (one of the criteria for due care).

### Frequency of Aftercare

The majority of physicians had had one (52.8%) or more (24.4%) aftercare conversation(s) with bereaved relatives of patients who died after EAS, while 22.8% did not have any aftercare conversation (Table 2). Among cases where the physician did have at least one aftercare conversation (n = 96), six relative(s) were referred to additional care. This involved referral of bereaved relative(s) to a psychological or spiritual caregiver to address emotional distress (including grief) and assist in coping with the loss of their loved one.

**TABLE 2:** Number of physicians who provided aftercare to bereaved relatives of patients who received euthanasia or physician-assisted suicide (N = 127) (Fourth evaluation of the Dutch euthanasia act, Netherlands, 2022).

	N (%)
Aftercare conversation with relatives	
Yes, once	67 (52.8)
Yes, multiple times	31 (24.4)
No	29 (22.8)
Among physicians who had at least one aftercare conversation	*N = 98*
Topics of aftercare conversations^a^	
Looking back on practical aspects of the EAS trajectory	51 (62.2)
Emotional experience of relatives during the EAS trajectory	41 (50.0)
Mental wellbeing of relatives following the EAS	19 (23.2)
Other	9 (11.0)
Aftercare conversation led to referral to additional care	
Yes	6 (6.3)
No	90 (93.8)

^a^ Open-ended question, of which the answers were categorized into four main categories (multiple answers possible).

Missings: topics 16, referral to additional care 2.

### Topics Aftercare Conversations

According to the physicians there were three main topics that were discussed in aftercare conversations: looking back on the practical aspects of the EAS trajectory (62.2%), the emotional experience of relatives during the EAS trajectory (50.0%), and the mental wellbeing of relatives following the EAS (23.2%) (Table 2). In most cases multiple topics were discussed in an aftercare conversation. Looking back on practical aspects of the EAS trajectory included both the period running up to the performance of EAS, the decision-making process as well as the performance of the EAS itself, as one physician wrote down:

“*The course of action during euthanasia and the legitimacy of the request.*”

This also sometimes included an evaluative component on how relatives, more practically, had experienced this trajectory, e.g., were they satisfied with how it went.

The emotional experience included how relatives emotionally experienced the EAS trajectory, e.g., the actual performance of the EAS:

“*Specifically, how the bereaved relatives had experienced the death of their loved one (and the manner in which they died). In this case, the experience was such for the bereaved relatives that they indicated that they would also want to die in this way in a similar situation.*”

Also, a few physicians wrote down that they themselves shared how they experienced the EAS.

Discussing the mental wellbeing of the bereaved relatives included discussing how the relatives were currently doing and how their grieving process went. Also some physicians wrote down that they had explored whether additional support was needed.

*“How the loved one experienced it, if she has any things she wants to discuss, if she has any questions, if she needs any help/support.”*

*“[How to] live without a partner after euthanasia.”*

Only two physicians wrote down the word “grief” in their response, while several other physicians used other words referring to grief (e.g., coping with the loss).

Finally, 11.0% of the physicians wrote down a number of other topics they discussed in aftercare conversations including: bereaved relatives expressing gratitude, reviewing the life of the patient, discussing issues that relatives experienced with the funeral director, and saying goodbye to each other after an intense period.

### Associations Between Aftercare Conversations and Patient-, Physician- and Process-Characteristics

Table 3 shows associations between patient-, physician- and process characteristics and having had a following up conversation (Table 3). The likelihood that physicians had an aftercare conversation with bereaved relatives was lower in cases of a patient without a (cohabiting) partner compared to patients with a cohabiting partner (OR 0.04–0.09) and for clinical specialists compared to general practitioners (OR 0.15). No other characteristics were significantly associated with having had an aftercare conversation.

**TABLE 3:** Associations between having had an aftercare conversation and patient, physician, and process characteristics (n = 127) (Fourth evaluation of the Dutch euthanasia act, Netherlands, 2022).

	Aftercare conversation with relatives (yes) (%)	Univariable OR (95% CI)	Multivariable OR (95% CI)
Patient characteristics			
Age			
<65 years (n = 17)	70.6	1.00	
65–79 years (n = 40)	77.5	1.44 (0.40–5.16)	
≥80 years (n = 65)	78.5	1.52 (0.46–5.04)	
Gender			
Male (n = 47)	76.6	1.00	
Female (n = 77)	77.6	1.00 (0.43–2.36)	
Partner			
Yes, cohabiting (n = 41)	95.1	1.00	1.00
Yes, not cohabiting (n = 8)	50.0	**0.05 (0.01–0.37)**	**0.04 (0.01–0.38)**
No, widow(er) (n = 63)	71.4	**0.13 (0.03–0.59)**	**0.09 (0.02–0.48)**
No, other (n = 13)	61.5	**0.08 (0.01–0.50)**	**0.07 (0.01–0.48)**
Diagnosis			
Cancer (n = 32)	81.3	1.00	
Dementia (n = 15)	86.7	1.50 (0.27–8.49)	
Accumulation of health problems related to old age (n = 52)	76.9	0.77 (0.26–2.31)	
Other diagnosis (n = 27)	66.7	0.46 (0.14–1.53)	
Level of dependency			
Independent (n = 27)	74.1	1.00	
Limited care dependent (n = 30)	76.7	1.15 (0.34–3.85)	
Care dependent (n = 69)	78.3	1.26 (0.45–3.54)	
Life expectancy			
<1 month (n = 31)	80.6	1.00	
1–5 months (n = 26)	69.2	0.54 (0.16–1.83)	
6–12 months (n = 24)	83.3	1.20 (0.30–4.84)	
>12 months (n = 45)	75.6	0.74 (0.24–2.28)	
Physician characteristics			
Medical specialty			
General practitioner (n = 90)	82.2	1.00	1.00
Clinical specialist (n = 13)	53.8	**0.25 (0.08–0.85)**	**0.15 (0.03–0.70)**
Elderly care physician (n = 23)	69.6	0.49 (0.18–1.40)	0.90 (0.29–2.81)
Gender^a^			
Male (n = 65)	76.9	1.00	
Female (n = 61)	77.0	1.01 (0.44–2.31)	
Age			
≤40 years (n = 24)	83.3	1.00	
41–50 years (n = 31)	67.7	0.42 (0.11–1.56)	
51–60 years (n = 44)	81.8	0.90 (0.24–3.37)	
>60 years (n = 27)	74.1	0.57 (0.14–2.26)	
Religious			
No (n = 92)	77.2	1.00	
Yes (n = 35)	77.1	1.00 (0.40–2.53)	
Work experience			
≤10 years (n = 14)	85.7	1.00	
11–20 years (n = 42)	71.4	0.42 (0.08–2.15)	
21–30 years (n = 46)	84.8	0.93 (0.17–5.08)	
>30 years (n = 25)	68.0	0.35 (0.06–1.97)	
Process characteristics			
Opinion of relatives about EAS request			
Neutral (n = 6)	50.0	1.00	
Supportive (n = 119)	78.2	3.58 (0.68–18.78)	
Not supportive (n = 2)	100.0	b	
Duration of EAS decision-making process			
<1 month (n = 74)	78.4	1.00	
1–3 months (n = 41)	73.2	0.75 (0.31–1.82)	
>3 months (n = 11)	81.8	1.24 (0.24–6.33)	
Euthanasia or physician assisted suicide			
Euthanasia (n = 124)	77.4	1.00	
Physician-assisted suicide (n = 3)	66.7	0.58 (0.05–6.67)	
Complications			
Yes (n = 6)	83.3	1.00	
No (n = 121)	76.9	0.66 (0.07–5.93)	

Missing values: age patient 5, gender patient 3, partner 2, diagnosis 1, level of dependency 1, life expectancy 1, medical specialty 1, age physician 1, duration of EAS decision-making process 1.

Bold odds ratios in univariable regression indicates a *p*-value of < 0.10, in multivariable regression it indicates a *p*-value of < 0.05.

^a^ There was one physician who indicated to have gender “other.” This is treated as a missing value in this analysis due to problems with statistical power.

^b^ Not included in analyses because all cases in which relatives were not supportive of the EAS request had had an aftercare conversation.

## Discussion

Providing aftercare to bereaved relatives of patients who died after EAS appears to be common practice among physicians in the Netherlands. Most physicians had at least one aftercare conversation with bereaved relatives, with GPs more frequently engaging in these conversations than clinical specialists. In addition, physicians were more likely to have an aftercare conversation with cohabiting partners of the patient than with other relatives. In these conversations physicians together with bereaved relatives looked back on the practical aspects of the EAS trajectory, discussed the emotional experience of relatives during the EAS trajectory and the mental wellbeing of relatives afterwards. Since no other case-specific characteristics were found to be associated with the provision of aftercare, it seems that whether bereaved relatives receive aftercare does not depend much on specific characteristics of the EAS trajectory. While aftercare conversations are common after EAS, they rarely lead to referrals for additional care, such as to spiritual caregivers or psychologists.

### Aftercare Provision Related to the Patient’s Partner Status and the Physician’s Medical Specialty

In cases of patients without a (cohabiting) partner it was less likely that the physician had had an aftercare conversation. An explanation may be that physicians may assume that partners are most affected by a loss and hence more inclined to have an aftercare conversation. Additionally, cohabiting partners may be more likely to accompany each other to doctor’s appointments. This increases the likelihood that physicians have already interacted with them, thereby reducing the barrier for initiating an aftercare conversation.

Furthermore, GPs were more likely to have an aftercare conversation compared to clinical specialists (82.2% vs 53.8%). The share of GPs that had aftercare conversations in our study is comparable to previous non-EAS research. Penders et al. showed that 93% of GPs engaged in aftercare conversations after non-sudden deaths in general. However, it must be noted that they also included plans for a follow-up conversation, possibly explaining the slightly higher prevalence of follow-up conversations [13]. Differences between GPs and clinical specialists regarding aftercare can be explained in various ways. Compared to clinical specialists, it is more likely that GPs treat multiple members from the same family, or have done so in the past, and therefore already know the bereaved relatives. This possible pre-existing relationship between the GP and the bereaved relative is likely to reduce the threshold for initiating an aftercare conversation [19].

In contrast, clinical specialists typically do not have a treatment relationship with relatives of a patient. It could be that clinical specialists therefore presume that aftercare for bereaved relatives is not their responsibility and that bereaved relatives will visit their GP in case of psychological problems, such as complicated grief. However, our findings suggest that aftercare conversations most often concern reviewing the EAS trajectory, which may be most appropriate with the physician who performed the EAS (e.g., clinical specialist). Two studies among bereaved relatives of ICU patients showed that they preferred to have an aftercare conversation with someone who had been closely involved with the case [7, 20]. In the case of EAS, this is most likely the physician who performed the EAS. It is therefore important that clinical specialists are also aware of the possible needs of bereaved relatives for an aftercare conversation after EAS.

Apart from the provision of aftercare being related to partner status and the type of physician involved, no other characteristics were associated. This shows that little selection is made in who is offered an initial aftercare conversation, which seems to be desirable. In contrast, additional aftercare, such as referral to a psychologist, may be specifically targeted for bereaved relatives who need this extra support [5].

Although most physicians provide aftercare, in approximately one in four EAS cases no aftercare is provided. The reasons for this are unknown. Bereaved relatives may not want or need aftercare [20], but there may also be barriers that hamper the provision of aftercare, e.g., administrative issues, work pressure or lack of education about bereavement (care) [19, 21]. It would be valuable to get insight into the reasons for not providing aftercare following EAS to overcome potential barriers and make aftercare after EAS accessible for everyone.

### Aftercare After EAS Should Not Only Focus on Grief

In contrast to the literature and guidelines on aftercare (practices) that frequently emphasize grief (e.g., [22–24]), physicians in our study indicated that aftercare conversations after EAS encompass a wide range of topics, with grief being just one of the topics. The predominant topics discussed by these physicians involve the practical aspects of the EAS trajectory and the emotional experiences during this trajectory. Assuming that the content of these conversations reflect the preferences of bereaved relatives, it suggests that relatives may not solely desire to address grief during aftercare. Future research into the preferences and needs of bereaved relatives regarding the provision of bereavement care by the deceased’s person GP would be very insightful. Although discussing mental wellbeing and in particular grief may not be one of the top priority preferences of relatives, physicians should not omit discussing this topic in aftercare conversations as it is known that individuals risk developing grief disorders and these people are less likely to seek support themselves [15]. The fact that physicians seem to rarely discuss grief in aftercare conversations following EAS may be (partially) due to their lack of formal education in grief and bereavement [24]. Training in aftercare and bereavement support could be highly valuable for physicians, who also indicated a need for such training themselves [25].

Previously, a tiered public health model for bereavement care has been proposed, recommending that individuals who are considered low risk for poor bereavement outcomes receive support from relatives instead of professionals [5, 23]. However, if bereaved individuals feel a need to discuss the practical aspects of the EAS trajectory, an initial aftercare conversation with a professional may need to extend beyond bereaved relatives at high risk of poor bereavement outcomes to encompass a broader group of relatives. This more low threshold type of contact with all relatives, as also recommended in the Dutch guideline *Grief in the palliative phase* [26], can serve as a gateway to identifying relatives at risk of developing psychological problems, such as prolonged grief disorder, who may need more specialized support.

While this study is based on the perspective of physicians, the needs and preferences of relatives’ regarding aftercare following EAS should also be explored in future research. In other end-of-life care practices bereaved relatives have indicated a need for looking back on the dying process and the trajectory leading up to the death. For instance, Milberg et al. (2008) found that bereaved relatives wanted to review what happened during the palliative phase in the palliative care unit [6]. Another recent study among bereaved relatives of ICU patients showed that the primary reasons for wanting an aftercare conversation were related to practical aspects of the ICU admission and a wish to exchange experiences with the physician involved in the trajectory leading up to the death of a patient, rather than to mental wellbeing [7]. Physicians should therefore be attentive to these aspects of aftercare, which already seems to be the case considering the topics of aftercare conversations reported by physicians in our study.

### Strengths and Limitations

This study has some strengths and limitations. As no quantitative data are currently available about aftercare after EAS this study does provide valuable insights into the current practices. Moreover, with an increasing number of countries legalizing EAS, these findings also have international relevance. It would be valuable to also gain more insight into why approximately a quarter of GPs did not provide aftercare to relatives of their patients. This can highlight certain barriers that may exist for the provision of aftercare.

The sample of physicians in this study is relatively small, possibly resulting in limited power in the analyses. In addition, since the data about the topics of the aftercare conversation were derived from a questionnaire and not from an interview, we could not ask physicians for further clarification. We recommend to conduct a qualitative interview study to gain a deeper understanding of aftercare after EAS (e.g., to whom is it provided, when is it provided, why it is provided), which also includes the perspectives of bereaved relatives.

### Conclusion

Most physicians have at least one aftercare conversation with bereaved relatives after EAS, and in a few cases this leads to referral to additional care. The aftercare conversations cover amongst others practical aspects of the EAS trajectory, emotional experiences during the EAS trajectory, and mental wellbeing after death. Such aftercare conversations with a physician are likely to be valuable for all bereaved relatives, and not just for “at risk” populations typically targeted by policies and guidelines. Conversations about the performed EAS and relatives’ wellbeing can be low threshold, and therefore likely feasible not only for GPs but also for other physicians. To improve the quality of aftercare, future research should explore the experiences and needs of bereaved relatives regarding aftercare following EAS.

## Data Availability

The dataset used and/or analyzed during the current study is available from the corresponding author upon reasonable request.
