# Supplementary material for: Aftercare Provision for Bereaved Relatives Following Euthanasia or Physician-Assisted Suicide: A Cross-Sectional Questionnaire Study Among Physicians
Source: Int J Public Health. 2024 Jul 25;69:1607346. doi: 10.3389/ijph.2024.1607346 (PMC11306013; doi:10.3389/ijph.2024.1607346)

**Additional file 2**

If physicians only had experience with an EAS request of a person with dementia or both dementia and another condition (not an accumulation of health problems related to old age), they were directed to the version on dementia. If physicians only had experience with an EAS request of a person with an accumulation of health problems related to old age or both an accumulation of health problems related to old age and another condition (not dementia), they were directed to the version on an accumulation of health problems related to old age. If physicians had both experience with an EAS request of a person with dementia and of a person with an accumulation of health problems related to old age, they were randomly assigned to the version on dementia or the version on an accumulation of health problems related to old age. If physicians only had experience with an EAS request of a person with another condition and not with dementia and an accumulation of health problems related to old age, they were directed to the version on another condition.


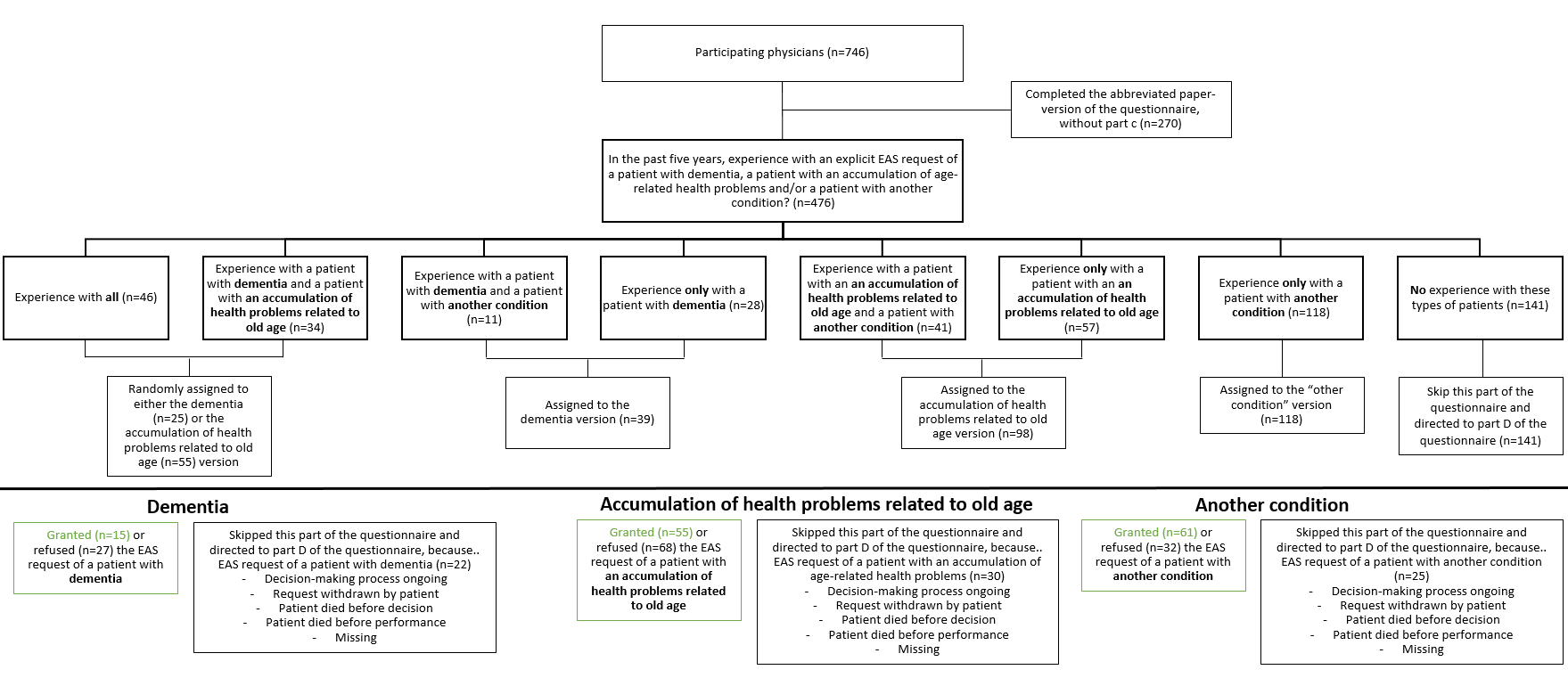

Supplement: Supplementary file 1 [file DataSheet2.docx]
